# Anomaly Detection in Nuclear Power Production Based on Neural Normal Stochastic Process

**DOI:** 10.3390/s25144358

**Published:** 2025-07-12

**Authors:** Linyu Liu, Shiqiao Liu, Shuan He, Kui Xu, Yang Lan, Huajian Fang

**Affiliations:** 1 China Nuclear Power Operation Technology Corporation, Wuhan 430233, China; liuly01@cnnp.com.cn (L.L.); liusq02@cnnp.com.cn (S.L.); heshuan@cnnp.com.cn (S.H.); lanyang@cnnp.com.cn (Y.L.); fanghj01@cnnp.com.cn (H.F.); 2 College of Nuclear Science and Technology, Naval University of Engineering, Wuhan 430033, China

**Keywords:** nuclear power production, anomaly detection, incomplete time series, neural normal stochastic process

## Abstract

To ensure the safety of nuclear power production, nuclear power plants deploy numerous sensors to monitor various physical indicators during production, enabling the early detection of anomalies. Efficient anomaly detection relies on complete sensor data. However, compared to conventional energy sources, the extreme physical environment of nuclear power plants is more likely to negatively impact the normal operation of sensors, compromising the integrity of the collected data. To address this issue, we propose an anomaly detection method for nuclear power data: Neural Normal Stochastic Process (NNSP). This method does not require imputing missing sensor data. Instead, it directly reads incomplete monitoring data through a sequentialization structure and encodes it as continuous latent representations in a neural network. This approach avoids additional “processing” of the raw data. Moreover, the continuity of these representations allows the decoder to specify supervisory signals at time points where data is missing or at future time points, thereby training the model to learn latent anomaly patterns in incomplete nuclear power monitoring data. Experimental results demonstrate that our model outperforms five mainstream baseline methods—ARMA, Isolation Forest, LSTM-AD, VAE, and NeutraL AD—in anomaly detection tasks on incomplete time series. On the Power Generation System (PGS) dataset with a 15% missing rate, our model achieves an F1 score of 83.72%, surpassing all baseline methods and maintaining strong performance across multiple industrial subsystems.

## 1. Introduction

Nuclear power generation operates in a more complex and extreme production environment compared to conventional energy sources. To ensure safe electricity production, nuclear power plants deploy a large number of sensors to monitor various physical indicators during production, enabling early detection of anomalies [1,2]. As a result, anomaly detection has become a key task in ensuring the safety of nuclear power plants. The goal of anomaly detection is to timely identify abnormal or unusual data patterns sent by sensors, enabling effective intervention and ensuring the normal operation of the system [3,4,5].

In real-world nuclear power monitoring, incomplete time series due to sensor failures or transmission issues present significant challenges to traditional anomaly detection. These models either ignore the missingness or require preprocessing via imputation, which can obscure anomalies and introduce bias. High ionizing radiation can lead to sensors being in abnormal states, negatively affecting sample collection, data transmission, and storage, resulting in incomplete samples [6]. The safe operation of nuclear power plants depends on high-quality data as the basis for process control decisions. Using incomplete samples for anomaly detection often leads to misdiagnosis or missed detection [7,8]. Therefore, performing anomaly detection on operating conditions under incomplete sensor data conditions has become a major challenge in the field of nuclear power plant anomaly detection.

The core problem this study addresses is how to perform effective anomaly detection directly on incomplete time series data, without relying on prior imputation. This is essential to reduce latency, preserve temporal uncertainty, and avoid noise from artificial data completion.

**Figure 1:**
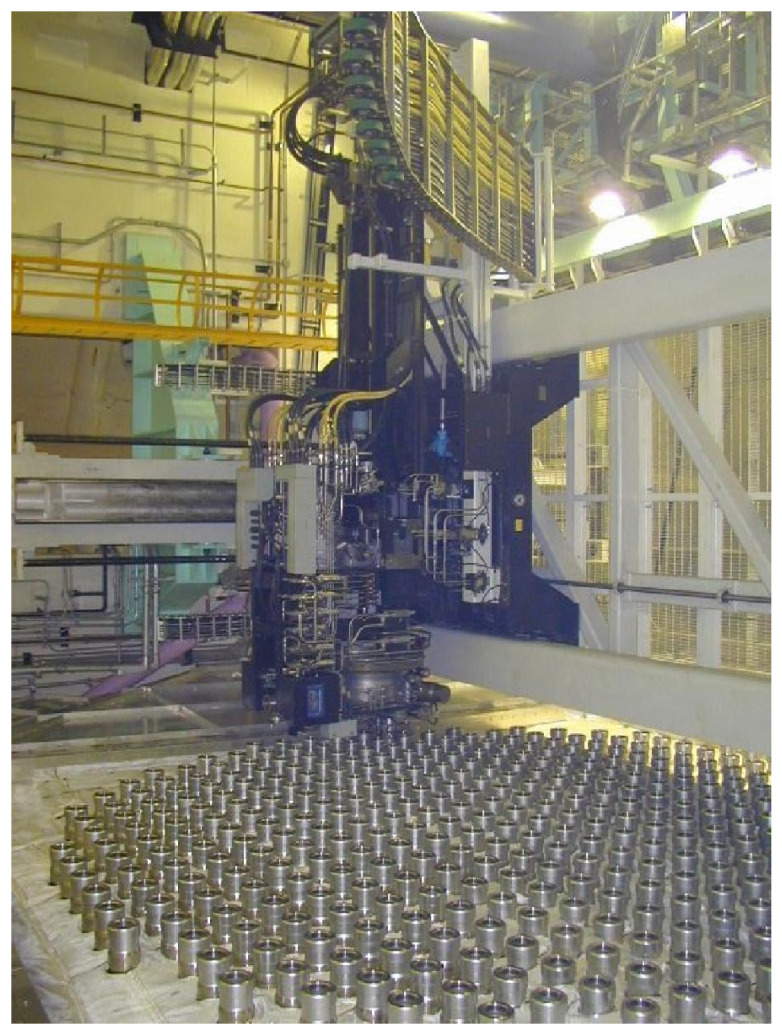
The internal structure of a nuclear reactor is densely populated with a wide range of sensors operating under harsh physical and environmental conditions. These factors frequently lead to incomplete data due to sensor failure, calibration drift, or maintenance interruptions, posing major challenges to downstream monitoring and anomaly detection tasks.

As shown in Figure 1, the internal structure of a nuclear reactor is densely populated with a wide range of sensors operating under harsh physical and environmental conditions. These factors frequently lead to incomplete data due to sensor failure, calibration drift, or maintenance interruptions, posing major challenges to downstream monitoring and anomaly detection tasks.

Nuclear power operation data is characterized by large volume, nonlinearity, and high dimensionality. As a result, various unsupervised learning-based algorithms play a crucial role in anomaly detection tasks [9]. In recent years, Seq-to-Seq models based on deep neural networks [10,11] and attention mechanisms [12,13] have demonstrated strong capabilities in modeling complex temporal dependencies. However, these methods often rely on the assumption that input data is complete and well-aligned over time—an assumption that is rarely satisfied in nuclear power production environments. While such methods focus on learning high-quality representations from fully observed data, they often overlook the challenges posed by sensor failures, asynchronous sampling, and partially missing sequences that are common in real-world industrial scenarios [14]. These limitations reduce the practical applicability of existing approaches, especially in safety-critical environments such as nuclear power plants. In particular, sensor sample loss can disrupt the temporal structure of input data and impair model performance [15].

Currently, data imputation techniques are widely adopted to address anomaly detection tasks involving missing values in time series. Common approaches include mean imputation [16], regression-based imputation [17], and KNN imputation [18]. However, mean imputation often distorts the prior distribution of high-dimensional data and is generally unsuitable for industrial anomaly detection. Regression imputation relies on the correlation between missing and observed features and requires prior knowledge that may not be available in practice, making it difficult to integrate into end-to-end neural network models. KNN imputation performs better when the missing ratio is high, yet in nuclear power monitoring scenarios, the missing rate is typically low, rendering KNN less effective [9]. Some methods leverage generative models such as GANs to synthesize missing data and then apply anomaly detection techniques on the completed time series. These methods partially mitigate the negative impact of missing data but may disrupt the intrinsic structure of the original observations.

We argue that although missing sensor values can degrade the local shallow semantic structure of time series data and hinder end-to-end training, their impact on higher-level latent semantics is more limited. Therefore, the focus of anomaly detection should shift from preserving local data completeness to capturing abstract semantic patterns that are robust to missing observations.

To address the challenge of performing anomaly detection on incomplete nuclear power monitoring data without relying on imputation, this paper proposes a novel approach that directly models incomplete sequences in the latent space. In this paper, we propose a novel method called the Neural Normal Stochastic Process (NNSP), which directly trains on incomplete nuclear power sensor time series without any imputation. To highlight the technical contributions of our work, we summarize the core innovations of NNSP as follows:

- Serialization embedding of incomplete samples: We design a framework that transforms incomplete sensor data into ordered sequence matrices and incorporates timestamp information through an independent input stream. This enables the model to extract temporal dependencies even in the presence of missing values.
- Neural Normal Stochastic Process representation: In the latent space, we construct a neural representation of a normal stochastic process that continuously interpolates incomplete series, allowing the decoder to operate seamlessly across missing regions.
- Decoder guided by future information: The decoder receives inputs sampled from the future distribution of the latent process, which implicitly forces the model to reconstruct forward-looking patterns and thus improves the timeliness of anomaly detection.
- The remainder of this paper is organized as follows. Section 2 reviews the related work on anomaly detection for time series with missing data. Section 3 introduces the proposed Neural Normal Stochastic Process (NNSP), detailing its encoder–decoder architecture and how it handles incomplete input. Section 4 presents experimental evaluations conducted on real-world nuclear monitoring datasets, including baseline comparisons, ablation studies, and parameter analysis. Section 5 concludes the study with a summary of contributions and potential directions for future work.

## 2. Related Work

Anomaly detection techniques based on sample reconstruction methods have seen significant improvements in performance. The principle of this approach is to allow the model to learn the hidden features of samples and then reconstruct new input samples based on the learned knowledge. When the reconstruction error exceeds a set threshold, the model determines that the input sample is anomalous [19]. InterFusion is a hierarchical neural network that serves as the backbone network, performing anomaly detection by reconstructing the dependencies of time series data [20]. Compared to NNSP, which learns latent semantic patterns directly from incomplete data without reconstruction, InterFusion requires complete sequences and focuses on explicitly rebuilding observed dependencies.

OmniAnomaly is also a reconstruction model, but instead of directly reconstructing samples, it generates reconstruction probabilities in the latent space of the neural network and uses these probability values as criteria for anomaly detection [21]. Unlike OmniAnomaly, which still relies on latent reconstructions derived from full input data, NNSP does not depend on complete sequences and performs prediction directly in the latent space without explicit reconstruction targets.

The Variational Autoencoder (VAE) is the core framework of several reconstruction models. It uses variational inference to identify the distribution of input samples and performs sampling based on this distribution to reconstruct data with the same distribution [22]. After being trained with normal samples, VAE is highly sensitive to the distribution patterns of anomalous samples, making it well-suited as a pattern recognition framework for anomaly detection. In contrast, NNSP avoids reconstructing the original input space entirely and instead reconstructs a forward-shifted latent representation, making it more robust to partial observability and missing data.

Additionally, the Generative Adversarial Network (GAN) has been widely applied to anomaly detection tasks [12,13,14]. This model is capable of accurately reconstructing samples and, in the process, separates normal and anomalous patterns as much as possible [9]. In contrast to GAN-based approaches that emphasize sample reconstruction quality, NNSP focuses on whether the latent process captures the temporal probability distribution of the data, enabling effective detection even without imputing or regenerating missing observations.

The degree of separation between these different patterns determines the model’s detection performance. The main difference between NNSP and GAN is that NNSP does not focus on sample reconstruction accuracy or data imputation accuracy, but rather on whether the high-level abstract representation of samples can correctly capture the probability distribution of the data at each time point. Throughout the reconstruction and detection process, no interpolation or intervention is performed on the incomplete raw data, which maximizes the accuracy of the sensor information.

## 3. Proposed Methods

In this work, we consider missing data as a representative and practically significant type of input error, which frequently occurs in nuclear power monitoring systems due to sensor degradation or communication faults. The proposed method is specifically designed to address this form of error without relying on imputation.

We propose NNSP that directly reads incomplete nuclear power production sensor data through a sequential structure and encodes it into continuous data patterns in the latent space. The decoder of NNSP samples the data pattern at the specified time from the Neural Normal Stochastic Process and generates reconstructed samples of the input at future time points based on the sampling information. Finally, the presence of anomalies in the input sample for the given time interval is determined by calculating the difference between the reconstructed sample and the original sample.

### 3.1. Sequentialization of Nuclear Power Monitoring Data

In nuclear power monitoring systems, gateways typically collect data streams sent by a set of sensors, with each sensor responsible for collecting one attribute. The values of these attributes change over time, forming a multivariate time series in the gateway, which can be formally represented as follows:

(1) $$Y=\left[\begin{array}{cccc} y_{1}^{1} & y_{2}^{1} & \ldots & y_{N}^{1}\\ y_{1}^{2} & y_{2}^{2} & \ldots & y_{N}^{2}\\ \vdots & \vdots & \ddots & \vdots \\ y_{1}^{m} & y_{2}^{m} & \ldots & y_{N}^{m} \end{array}\right]=\left[y\left(1\right),y\left(2\right),\ldots ,y\left(N\right)\right]\in R^{m\times N}.$$

*Y* represents the matrix form of the multivariate time series, where the element $y_{k}^{j}$ represents the value of the *j*-th attribute at the *k*-th time point. To simplify the representation, we can express it in vector form, where the elements *y_k_* represent the m-dimensional attribute vector of the multivariate time series at the *k*-th time point, containing all m attributes. In practice, missing data often occurs in nuclear power sensor data, so we modify Equation (1) as follows:

(2) $$Y\left(t\right)=\left[\begin{array}{ccccc} y^{1}\left(t_{1}\right) & \ldots & y^{1}\left(t_{k}\right) & \ldots & y^{1}\left(t_{n}\right)\\ y^{2}\left(t_{1}\right) & \ldots & y^{2}\left(t_{k}\right) & \ldots & y^{2}\left(t_{n}\right)\\ \vdots & \ddots & \vdots & \ddots & \vdots \\ y^{m}\left(t_{1}\right) & \ldots & y^{m}\left(t_{k}\right) & \ldots & y^{m}\left(t_{n}\right) \end{array}\right]=\left[y\left(t_{1}\right),\ldots ,y\left(t_{k}\right),\ldots ,y\left(t_{n}\right)\right]\in R^{m\times n}.$$

In Equation (2), *Y*(*t*) denotes the time series after it has been sequenced. Its corresponding original series can either be complete or have missing elements. The length of time of the record of the series is *n* (*n* <= *N*), and the dimension is *m*. *t_k_* denotes the real timestamp corresponding to the *k*-th observation. The element *y^j^*(*t_k_*) in the matrix *Y*(*t*) denotes the observation of the *j*-th sensor at the *tk*-th moment. It can be found that each column of *Y*(*t*) is a temporally ordered list of monitored attribute values. This sequential arrangement allows for non-equal time intervals between samples. Even if data at specific time points or time intervals are not recorded, this sequential structure can still ensure the structural integrity of the input sequence. The sequentialization method ensures that samples only need to be continuously input into the convolution window in order; regardless of how the actual time interval between adjacent samples changes, no vacant positions will appear in the convolution window. Also, we can select the independent variables in each column of *Y*(*t*) and construct a corresponding timestamp vector:

(3) $$T=\left(t_{1},\ldots ,t_{k},\ldots ,t_{n}\right)\in R^{n},$$

where *T* denotes the timestamp vector and *t_k_* denotes the *k*-th sample observation.

### 3.2. The Framework of Neural Normal Stochastic Process

The role of NNSP is to perform anomaly detection on time series under conditions of missing data. Its input port is a one-dimensional convolutional neural network, capable of reading data serialized through Equation (2). The input information is encoded into the latent space and represented using a Neural Normal Stochastic Process. The Neural Normal Stochastic Process does not depict the distribution of the original time series but describes the representation of the time series in the latent space and the stability and changes in this representation. As the semantic level in the latent space is high, partial missing of the original data can hardly shake the characteristics of the latent distribution but will only increase the instability of the latent distribution.

**Figure 2:**
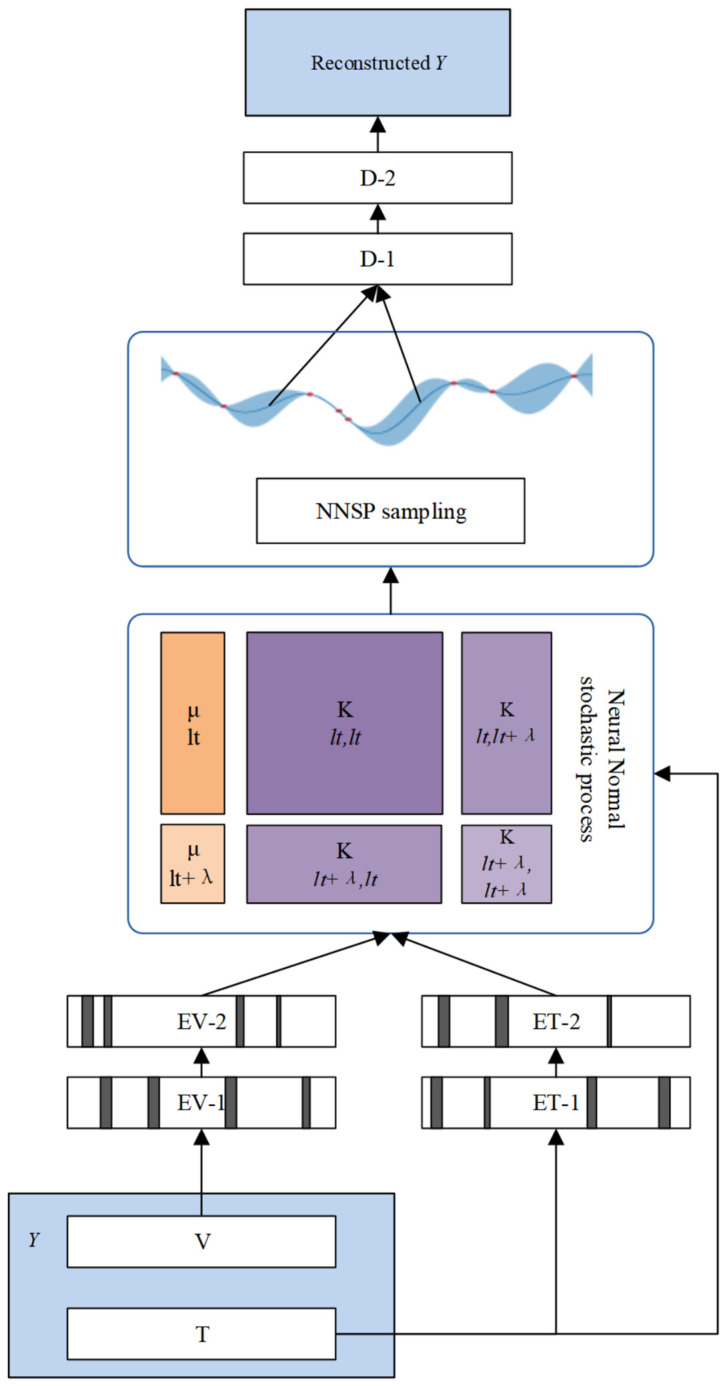
The framework of Neural Normal Stochastic Process.

As shown in Figure 2, the main structure of NNSP can be divided into three major parts. The first part is the input layer “reconstruction *Y*” and reconstruction layer “reconstruction *Y*”, located at both ends of NNSP, responsible for the input and reconstruction of time series information. The second part consists of the encoder *EV-X* and decoder *D-X*. They are a pair of fully connected neural networks with symmetrical mirror structures, encoding and decoding time series features, respectively. The third part is the latent space, which uses a Neural Normal Stochastic Process to represent the compressed information output by the encoder as continuous virtual observations. NNSP slides and samples the Neural Normal Stochastic Process according to the query time, obtaining a series of normal distributions related to time semantics. Finally, it samples the normal distribution and passes the sampling results into the decoder for decoding and reconstruction.

### 3.3. Encoding

In this study, we adopt a one-dimensional convolutional neural network (1D-CNN) as the encoder for multivariate time series, rather than using recurrent models such as LSTM or GRU. While recurrent architectures are effective in modeling long-range dependencies within complete sequences, our task focuses on detecting anomalies under incomplete data conditions, where local continuity is more critical. In this context, 1D-CNN is well suited for capturing temporal patterns within fixed receptive fields and sliding over serialized inputs, demonstrating greater robustness to missing entries. Moreover, 1D-CNN offers high computational efficiency and parallelism, which are essential for real-time analysis of high-frequency monitoring data in large-scale industrial settings. These advantages make it a more appropriate choice for preserving structural continuity in partially observed sequences without relying on recurrent dependencies that may be disrupted by missing data.

Based on these considerations, the input layer of NNSP includes a 1D-CNN that convolves the sequenced multivariate time series along the time axis, generating a feature map sequence (FMS). This process can be expressed as follows:

(4) $$f_{i}=\frac{1}{n}\sum_{k=1}^{n}\omega_{k}\left(Y{\left(t\right)}_{\left[:,i-s+1\right]}*F_{k}\left(s\right)\right),$$

where *f_i_* denotes the *i*-th feature of the feature map sequence. *Y*(*t*) represents the sequenced time series as defined by Equation (2). Since the time information is separated, the samples only need to enter the convolution window in sequence. Even if there is missing information, no null values will appear in the convolution window. *F_k_* (*s*) denotes the *k*-th 1D convolution kernel with window width *s*. In order to extract sufficient information from the multivariate time series, the convolution kernel is usually set to *n* (*n* > 1), and the asterisk ∗ refers to the convolution operation symbol, which performs the convolution operation on the left and right matrices. *i* denotes the serial number of the time series covered by the rightmost end of the convolution kernel, *s* denotes the window width of the convolution kernel, and *ω_k_* denotes the weight of the kth convolution kernel. When the convolution kernel sweeps through a time series of length *n*, a sequence of feature maps of length *n* − *s* + 1 can be obtained as follows:

(5) $$FMS=\left[f_{1},f_{2},\ldots ,f_{n-s+1}\right].$$

As shown in Equation (5), *FMS* denotes a feature map sequence, and *f_i_* denotes the *i*-th feature of the feature map sequence. Each element of the *FMS* is passed sequentially into the encoder for encoding. In order to extract the high-level semantics of the time series, the encoder contains two layers of fully connected neural networks, and the number of their neurons decreases sequentially so that the dimensionality of the elements in the *FMS* is further compressed, and finally, a set of hidden layer vectors is output in the latent space. The encoder is operated as follows:

(6) $$\begin{array}{l} H_{i}=U_{2}\left[w_{FC-2}\cdot U_{1}\left(w_{FC-1}\cdot FMS+b_{FC-1}\right)+b_{FC-2}\right]\\ =\left[h_{1},h_{2},\ldots ,h_{\dim\left(FC-2\right)}\right] \end{array}.$$

In Equation (6), dim(*FC* − 2) denotes the number of neurons in the second fully connected layer, which determines the dimensionality of the final latent vector. *H_i_* denotes the hidden layer vector obtained after the *i*-th section of the *FMS* is encoded, *W_FC_*_−1_ and *W_FC_*_−2_ denote the parameter matrices of the first and second fully connected neural networks, respectively, and *U*_1_ and *U*_2_ refer to the tanh activation function. The element *h_j_* denotes the *j*-th component of *H_i_*. It should be noted that *FC* − 1 and *FC* − 2 represent these two layers of fully connected neural networks, respectively, and their number of neurons decreases sequentially and satisfies *n* − *s* + 1 >= dim(*FC* − 1) > dim(*FC* − 2). This dimensionally compressed mapping filters out the preliminary information of the input sequence and reduces the number of parameters in the latent space while increasing the information density in the hidden layer. The vector *H_i_* is passed into latent space and then converted into continuous virtual observations by Neural Normal Stochastic Process.

### 3.4. Normal Stochastic Process Sampling

The stochastic component in NNSP plays a critical role in modeling uncertainty and enhancing robustness to missing data. By representing the latent space as a continuous-time normal stochastic process, the model captures not only the observed temporal dynamics but also infers the distribution of unobserved or future values. This probabilistic modeling enables the decoder to sample from latent regions that correspond to missing or uncertain segments, rather than relying on deterministic reconstruction. As a result, NNSP can maintain continuity in latent representations, reduce overfitting to incomplete input, and improve anomaly detection performance by considering the likelihood of future patterns rather than only observed values.

NNSP encodes the information of the hidden layer vector *H_i_* in latent space, which generates a Neural Normal Stochastic Process that unfolds along continuous time. Latent space is the highest density representation vector space of NNSP, meaning it has the relatively lowest vector dimension. This property keeps the matrix operations in latent space at a relatively small parameter scale. The input information of latent space includes, in addition to the hidden layer vector *H_i_*, the timestamp vector *T*. *H_i_* is the representation vector of a time series of length *n* in latent space, and its time dimension is compressed from n to dim(*FC* − 2), so the length of the corresponding timestamp vector *T* needs to be compressed in the same proportion.

(7) $$lt_{i-n+\dim\left(FC-2\right)}=T_{\left[i\right]}*FLT\left[n-\dim\left(FC-2\right)+1\right].$$

In Equation (7), $lt_{i-n+\dim\left(FC-2\right)}$ denotes the middle *i* − *n* + dim(*FC* − 2) component of *T* that is compressed, and *FLT* is a convolution kernel that takes the arithmetic mean on the time region it covers. In order to make the dimension of the *T* vector compressed to dim (*FC* − 2), the length of *FLT* needs to be set to *n*-dim(*FC* − 2) + 1; *T*[*i*] denotes the sequence of time points covered by the right boundary of *FLT*, which indicates the convolution operation. When *FLT* performs the convolution operation on the timestamp sequence *T* of length *n*, a hidden timestamp sequence of length dim(*FC* − 2) can be generated as follows:

(8) $$LT=\left(lt_{1},\ldots ,lt_{k},\ldots ,lt_{\dim\left(FC-2\right)}\right)\in R^{\dim\left(FC-2\right)}.$$

In Equation (8), *LT* denotes the hidden timestamp sequence, and *lt_k_* denotes the *k*-th of these sample observations. *h_i_* and *LT* can be regarded as abstract representations of the sequence basis vector and the time basis vector in the latent space, respectively, and can be tensed into a Neural Normal Stochastic Process $GP\left[\mu \left(lt\right),K\left(lt,lt\right)\right]$ that unfolds in the time dimension by an appropriate linear transformation and have

(9) $$z\left(lt\right)\sim NSP\left[\mu \left(lt\right),K\left(lt,lt\right)\right].$$

In Equation (9), *z*(*t*) denotes the probability distribution of the abstract representation in the latent space at moment *t*; $NSP\left[\mu \left(lt\right),K\left(lt,lt\right)\right]$ denotes a Neural Normal Stochastic Process whose mean function is *µ*(*lt*) and covariance function is *K*(*lt*,*lt*). A Normal Stochastic Process is defined on latent time *lt*, which can be regarded as a function of the normal distribution density function. Using this property, as long as a Normal Stochastic Process is constructed in latent space, NNSP can take the observation at any point on the continuous latent time and sample the normal distribution corresponding to this observation. According to Equation (9), the Neural Normal Stochastic Process is completely determined by its mean function *µ*(*lt*) and covariance function *K*(*lt*,*lt*). They can be constructed from the mean vector in the latent space with the kernel function matrix *K* with covariance properties. After translating some distance into the future, we take the multivariate normal distribution of each sample time point *lt_i_* in this Neural Normal Stochastic Process:

(10) $$\left[\begin{array}{c} z\left(lt\right)\\ z\left(lt+\lambda \right) \end{array}\right]\sim GP\left(\left[\begin{array}{c} \mu \left(lt\right)\\ \mu \left(lt+\lambda \right) \end{array}\right],\left[\begin{array}{cc} K\left(lt,lt\right) & K\left(lt,lt+\lambda \right)\\ K\left(lt+\lambda ,lt\right) & K\left(lt+\lambda ,lt+\lambda \right) \end{array}\right]\right).$$

In Equation (10), *z*(*lt*) denotes the distribution of the representation vector in latent space corresponding to time *lt*, and *λ* is the latent time translation distance. We can obtain the following theorem from this joint probability distribution.

**Theorem** **1.** *For a multivariate normal distribution $Z=\left[\begin{array}{c} z\left(lt\right)\\ z\left(lt+\lambda \right) \end{array}\right]$, its conditional distribution $z\left(lt+\lambda \right)|z\left(lt\right)$ also obeys a normal distribution:*

(11) $$\begin{array}{l} z\left(lt+\lambda \right)|z\left(lt\right)\sim \\ N\left(\begin{array}{l} \mu \left(lt+\lambda \right)+K\left(lt+\lambda ,lt\right)\cdot K^{-1}\left(lt,lt\right)\cdot \left[z\left(lt\right)-\mu \left(lt\right)\right],\\ K\left(lt+\lambda ,lt+\lambda \right)-K\left(lt+\lambda ,lt\right)\cdot K^{-1}\left(lt,lt\right)\cdot K\left(lt,lt+\lambda \right) \end{array}\right) \end{array}.$$

**Proof.**  Consider the covariance function matrix $\Sigma =\left(\begin{array}{cc} K\left(lt,lt\right) & K\left(lt,lt+\lambda \right)\\ K\left(lt+\lambda ,lt\right) & K\left(lt+\lambda ,lt+\lambda \right) \end{array}\right)$.

Let $lt_{1}=lt,\;lt_{2}=lt+\lambda$ and $\mu \left(lt\right)=\mu_{1},\mu \left(lt+\lambda \right)=\mu_{2}$; the inverse matrix of Σ is

$$\Sigma^{-1}=\left(\begin{array}{cc} K^{-1}\left(lt_{1},lt_{1}\right)\left[E+K\left(lt_{1},lt_{2}\right)A^{-1}K\left(lt_{2},lt_{1}\right)K^{-1}\left(lt_{1},lt_{1}\right)\right]\;, & -K^{-1}\left(lt_{1},lt_{1}\right)K\left(lt_{1},lt_{2}\right)A^{-1}\\ -A^{-1}K\left(lt_{2},lt_{1}\right)K^{-1}\left(lt_{1},lt_{1}\right), & A^{-1} \end{array}\right),$$

where $A=K\left(lt_{2},lt_{2}\right)-K\left(lt_{2},lt_{1}\right)\cdot K^{-1}\left(lt_{1},lt_{1}\right)\cdot K\left(lt_{1},lt_{2}\right)$, and *E* denotes the unit matrix of the same shape so that the Mahalanobis distance can be obtained as follows:

(12) $$\mu \left(lt_{2}|lt_{1}\right)=\mu^{*}=\mu_{2}+K\left(lt_{2},lt_{1}\right)K^{-1}\left(lt_{1},lt_{1}\right)\left[z\left(lt_{1}\right)-\mu_{1}\right],$$

(13) $$\Sigma_{\left(lt_{2}|lt_{1}\right)}=A=K\left(lt_{2},lt_{2}\right)-K\left(lt_{2},lt_{1}\right)\cdot K^{-1}\left(lt_{1},lt_{1}\right)\cdot K\left(lt_{1},lt_{2}\right).$$

Substituting $lt_{1}=lt,\;lt_{2}=lt+\lambda$ and $\mu \left(lt\right)=\mu_{1},\mu \left(lt+\lambda \right)=\mu_{2}$ into Equations (12) and (13), we get

$$\begin{array}{l} z\left(lt+\lambda \right)|z\left(lt\right)\sim \\ \;\;N\left(\begin{array}{l} \mu \left(lt+\lambda \right)+K\left(lt+\lambda ,lt\right)\cdot K^{-1}\left(lt,lt\right)\cdot \left[z\left(lt\right)-\mu \left(lt\right)\right],\\ K\left(lt+\lambda ,lt+\lambda \right)-K\left(lt+\lambda ,lt\right)\cdot K^{-1}\left(lt,lt\right)\cdot K\left(lt,lt+\lambda \right) \end{array}\right) \end{array}.$$

□

Theorem 1 provides a specific calculation method for inferring *z*(*lt* + *λ*) using the latent distribution *z*(*lt*). That is, construct the conditional distribution $z\left(lt+\lambda \right)|z\left(lt\right)$ according to Equation (11), which follows a Normal Stochastic Process. Here, *μ* comes from the information of the encoded sample values, while the kernel function matrix *K* comes from the encoded sample time information. Although these samples may be incomplete, as long as there is input information and a time shift increment *λ*, the analytical expression of the Neural Normal Stochastic Process (a continuous family of normal distribution functions) with continuity properties can always be determined. In this process, the encoding information of incomplete samples achieves a continuous representation. Moreover, since *λ* can take any positive real number, the position of the sampling point in the future time can also slide continuously on the virtual observation.

(14) $$\mu_{j}=w_{\mu }h_{j}+b_{\mu },\;M=\left(\mu_{1},\mu_{2},\ldots ,\mu_{\dim\left(FC-2\right)}\right)\in R^{\dim\left(FC-2\right)}.$$

In Equation (14), *M* denotes the mean vector in the NNSP, and *h_j_* refers to the jth component in the hidden layer of the time series within the latent space components. *μ_j_* denotes the *j*-th mean in *M*, *w_u_* is the parameter matrix of the linear transformation, and *b_μ_* denotes the bias of the linear transformation, and they are all set as learnable parameters in latent space. For the kernel function matrix *K*(*t_i_,t_j_*), we use a normal kernel function to map it to infinite dimensions as follows:

(15) $$K\left(t_{i},t_{j}\right)=\sigma^{2}\exp\left(-{\left\|t_{i}-t_{j}\right\|}_{2}^{2}/2l^{2}\right).$$

In Equation (15), the covariance matrix *K* is defined with a normal kernel function, and *l* and *σ* are the two hyperparameters of the kernel function. After obtaining the mean vector *M* and the kernel function matrix *K*, we can use the reparameterization technique [22] to sample the Neural Normal Stochastic Process in latent space at the future time point *lt* + *λ*. Since each virtual observation point in the Neural Normal Stochastic Process is a normal distribution, we can determine the mean $\mu \left(lt_{2}|lt_{1}\right)$ and the covariance matrix of the observation points $\Sigma_{\left(lt_{2}|lt_{1}\right)}$ by Theorem 1.

(16) $$v\left(\lambda \right)=\mu \left(lt+\lambda |lt\right)+diag\left[\Sigma_{\left(lt+\lambda |lt\right)}\right]\cdot \eta ,\;\eta \sim N\left(0,1\right).$$

In Equation (16), the sampling code *v*(*λ*) is the output of latent space, which is the result of sampling the Neural Normal Stochastic Process in latent space at each observation point in the future *λ* using the reparameterization trick. *u*(*) represents the mean of the observation points, and Σ(*) represents the variance of the observation points. *diag*(*) denotes a new vector that takes the main diagonal elements of the matrix and arranges them by row number (or by column number). *η* denotes a random variable that obeys the standard normal distribution. Equation (16) separates the random sampling process of *v*(*λ*) and assigns it to *η* so that the gradient passed backward by the decoder during NNSP training can pass through *v*(*λ*) smoothly and update its internal parameters.

### 3.5. Anomaly Detection

The decoder of NNSP is responsible for decoding *v*(*λ*), which consists of two layers of fully connected neurons that are structurally mirror symmetric to the encoder. The reconstruction layer uses the deconvolution neural network to reconstruct the decoded *v*(*λ*). Since *v*(*λ*) is a latent representation of the data with a latent time translation distance *λ*, the reconstructed information inherits this translation distance. It needs to be expanded back to its original length according to the compression ratio of the latent space when decoded from the latent space to correspond to the reconstructed time series. This correspondence can be expressed as follows:

(17) $$\lambda^{\prime }=\lambda \cdot \dim\left(T\right)/\dim\left(FC-2\right).$$

In Equation (17), $\lambda^{\prime }$ is the real-time translation distance, and dim(*T*) is the dimension of the timestamp vector *T*. $\lambda^{\prime }$ inherits the continuity of *λ*, so in the model training phase, it is only necessary to specify the length of *λ* to train $Y(t+\lambda^{\prime })$ as the supervised signal *Y*(*t*). We transform the loss function in VAE [22] to fit NNSP, where the loss is

(18) $$\begin{array}{c} l_{\left[y\left(t\right),\phi ,\theta \right]}=-D_{KL}\left[q_{\phi }\left(v\left(\lambda \right)|y\left(t\right)\right)||p_{\theta }\left(v\left(\lambda \right)\right)\right]+\\ E_{v\left(\lambda \right)\sim q\left[v\left(\lambda \right)|y\left(t\right)\right]}\left[\log p_{\theta }\left(y\left(t+\lambda^{\prime }\right)|v\left(\lambda \right)\right)\right] \end{array}.$$

In Equation (16), $l_{\left[y\left(t\right),\phi ,\theta \right]}$ denotes the loss function of NNSP, and *φ* and *θ* denote the encoder and decoder parameters, respectively. *D_KL_* denotes the *KL* scatter, and *q* denotes the output probability of the encoder. *p* denotes the output probability of the decoder. NNSP, by detecting the differences between reconstructed samples and real samples, assesses the occurrence of anomalies. It reconstructs the incomplete *Y*(*t*) to generate samples that represent virtual observation points. These reconstructed samples, along with the actual ones, form a collection of anomaly detection pairs, facilitated by a prediction function. The Mean Squared Error (MSE) serves as a metric to gauge the reconstruction error for these observation points. Anomaly alerts are triggered for time series data when the error surpasses a predefined threshold. The reconstruction error is defined as follows:

(19) $$MSE\left[Y\left(t+\lambda^{\prime }\right),\hat{Y}\left(t+\lambda^{\prime }\right)\right]=\frac{1}{\dim\left[Y\left(t+\lambda^{\prime }\right)\right]}{\left\|Y\left(t+\lambda^{\prime }\right)-\hat{Y}\left(t+\lambda^{\prime }\right)\right\|}_{2}^{2}>\alpha$$

In Equation (19), *MSE* (*) denotes the mean squared error, and *α* denotes the anomaly threshold, which can be set to a specific positive value depending on the specific industrial domain. In this paper, we set the anomaly threshold *α* to 0.211. This value was obtained through grid search on the training set. Among multiple candidate thresholds, *α* = 0.211 yielded relatively the best performance.

## 4. Experiments and Discussion

### 4.1. Experimental Settings

In this study, we used an anomaly state dataset from China Nuclear Power Operation Technology Corporation, with a time span from 4 March 2019 to 3 March 2021, to evaluate the performance of NNSP in anomaly detection tasks under incomplete sensor data conditions. In this system, the proportion of invalid sampling points for a single attribute to all samples is maintained within 10%. However, since most samples are high-dimensional time series composed of multiple attributes, when a single attribute value is not effectively collected, the monitoring system defines the entire high-dimensional sample point as missing, resulting in a higher actual proportion of data loss. The dataset was partitioned by category, with 80% of the samples used for training and the remaining 20% used for testing. To optimize the model, we adopted the Adam optimizer, a widely used and efficient optimization algorithm. The learning rate was set to 0.001, with the exponential decay rate for the first moment estimate (β_1_) set to 0.9 and the second moment estimate (β_2_) set to 0.999. The model was trained for 28,517 iterations until convergence. Additionally, to ensure the robustness and generalizability of the results, cross-validation was performed during the experimental process.

The power generation system (PGS) monitoring time series contains 12-dimensional attributes (temperature, turbine speed, pressure, etc.). It is a closed system with little external influence but extreme internal physical conditions. Its data loss pattern and anomaly pattern are usually closely related to production decisions and have strong periodicity. The power transmission system (PTS) monitoring gateway collects 15-dimensional time series (it contains micro-meteorological sensor data streams, tilt sensor data streams, etc.). It is a subsystem mainly affected by the external environment. The production equipment and detection equipment are distributed in a vast open physical space. Its equipment anomalies and data loss patterns are usually diverse, and the pattern stability is low. The power distribution system (PDS) sends back a seven-dimensional time series (instantaneous voltage, current, etc.). Similarly to PGS, PDS is also in a closed and stable physical environment, and with the change in environment and electricity demand, it shows strong periodicity. The equipment and sensors can work relatively stably. The transformer substation system (TSS) sends back a 10-dimensional time series (insulated gas sensors, temperature sensors, etc.). This system is also affected by internal and external factors. The corresponding equipment is relatively sensitive to changes in internal electromagnetic factors and external environmental factors, so its anomaly patterns usually show significant attribute dependence. The equipment assembly line (EAL) produces a 16-dimensional data stream.

The data was collected during the trial production stage. The sensors and monitored entities were all in a high-load test state, so the data Missing Rate and Anomaly Rate were both high. The Missing Rate is defined as the ratio of time steps where one or more feature values are missing, relative to the total number of time steps. The Anomaly Rate is calculated as the proportion of time steps labeled as anomalous in the dataset, relative to the total number of time steps. We evaluate model performance using standard metrics for unsupervised anomaly detection: precision, recall, and F1 score, which are also used in the baseline methods described in the Section 2.

The specifications of the five subsystems used in our experiments are summarized in Table 1. These include the Missing Rate, which reflects the proportion of invalid samples due to incomplete sensor readings, and the Anomaly Rate, which denotes the proportion of time steps labeled as abnormal. The length indicates the total number of time points collected for each system, while the frequency shows the sampling interval. These statistics illustrate the diversity and complexity of the monitoring scenarios.

The detailed specifications of the dataset are shown in Table 1.

### 4.2. Result Analysis and Discussion

We select five mainstream anomaly detection methods as the baseline for unsupervised learning. These include ARMA [23], Isolation Forest [24], LSTM-AD [25], VAE [26], and NeutraL AD [27]. These methods were selected to represent a diverse range of anomaly detection techniques: ARMA as a classical statistical model, Isolation Forest as a tree-based ensemble method, LSTM-AD and VAE as deep learning models, and NeutraL AD as a self-attention-based method. All are unsupervised and have been widely applied to time series anomaly detection tasks, including those with industrial and incomplete data. These baseline methods were selected in accordance with the approaches reviewed in the Section 2, ensuring consistency between theoretical references and experimental comparisons.

To ensure a fair comparison, we randomly apply interval masking to sample points in each dataset at a certain proportion, increasing the sample loss rate of each dataset to 15%.

As shown in Table 2, NNSP maintains leading F1 scores on all four datasets and maintains a precision and recall rate of about 80%, despite a 15% data missing rate, with corresponding F1 scores all over 0.8. Under normal circumstances, NeutraL AD, based on the self-attention mechanism, can achieve an F1 score close to 0.9 in multiple tasks. However, when data is missing, the advantage of NNSP becomes more apparent. We analyze this as NNSP uses the latent distribution of data to identify anomalies, ignoring specific local details and focusing more on the deep semantic distribution of input information. Even if data is missing locally, NNSP can reconstruct what it considers to be normal samples based on the distribution of the missing point’s context and the similar generative semantic distribution detected in history, thereby outputting anomaly alerts according to Equation (17).

**Figure 3:**
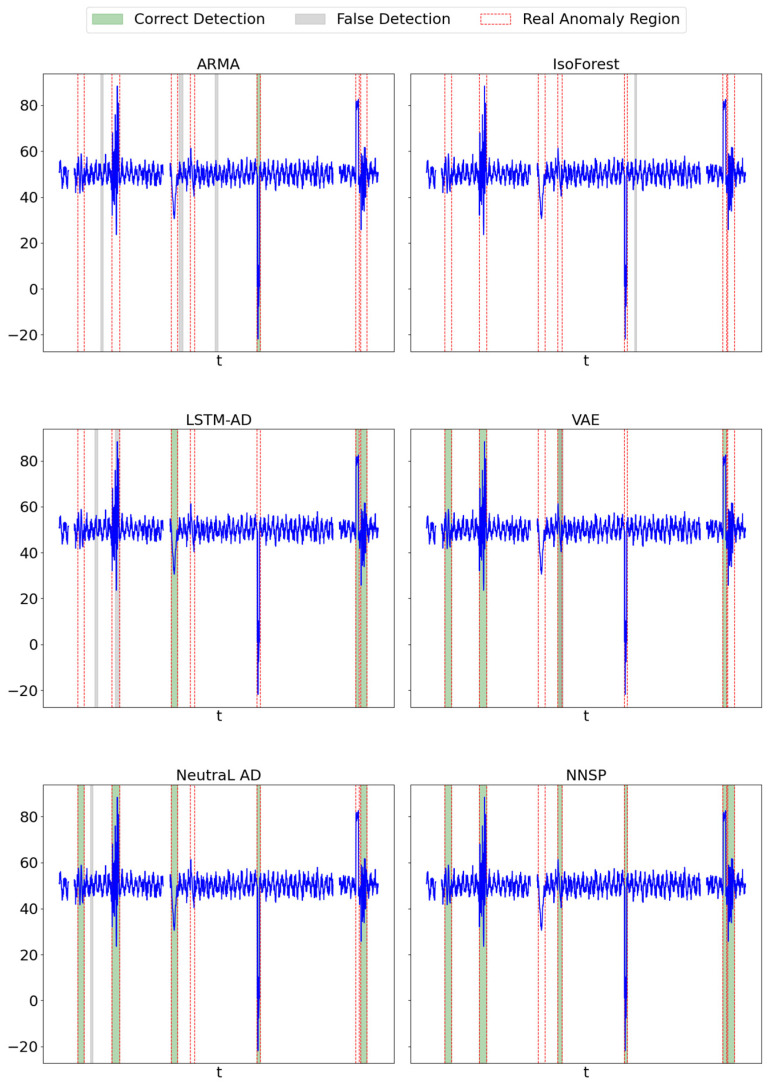
Anomaly detection performance.

Figure 3 visually demonstrates the anomaly detection performance of the five methods on one selected dimension of the PGS dataset. The horizontal axis represents relative time steps (i.e., sampling points), and the vertical axis shows the corresponding sensor values. All methods are evaluated on the same time interval to ensure comparability. Ground truth anomaly intervals are marked by shaded regions, while anomaly predictions from each method are indicated by colored markers. Although the visualization does not explicitly differentiate between true positives, false positives, and false negatives, readers can assess detection effectiveness by observing the alignment between predicted points and the shaded anomaly regions.

Among the methods, NNSP performs the best, with no false predictions and only one true anomaly interval missed. In contrast, its performance in the power distribution system (PDS) is relatively lower, which we attribute to the lower dimensionality of the PDS dataset. The reduced feature space provides less contextual information, making it more difficult for NNSP to reconstruct realistic future patterns. On the other hand, the power generation system (PGS) benefits from both high data dimensionality and a stable environment with strong periodicity, as it is less influenced by external factors. This allows NNSP to achieve the highest F1 scores even in the presence of missing data. The transformer substation system (TSS) shows the lowest F1 score. Although its data dimensionality is higher than PDS, the presence of strong external disturbances introduces irregular and unstable patterns, making it difficult for the model to distinguish between normal fluctuations and true anomalies. As a result, the model must balance internal and external anomaly patterns, which reduces its detection effectiveness.

We conduct ablation to explore the impact of components of NNSP on model performance. These include the sequential input structure (SIS), which is compatible with incomplete sequences, and the Neural Normal Stochastic Process (HGP), which extends sequences in a continuous time dimension. Additionally, we examine the Normal Stochastic Process Sampling (GPS) component for sampling time series with Normal Stochastic Processes. These components are individually removed during the experiment, and we assess the extent to which the model’s performance is affected. It is crucial to note that GPS is a component that inherits from HGP. When HGP is removed, the decoder cannot sample virtual observations. To maintain fairness in our evaluation, we replace virtual observation sampling with the resampling technique in variational inference, directly inputting samples from the distribution. In this configuration, GPS effectively becomes normal sampling (GS). The results are presented in Table 3, where “+” indicates the retention of the component, and “−” indicates its removal.

From Table 3, the model’s predictive power decreases by about 30% after removing the three components. In contrast, when the sequential input structure is applied alone, the model’s predictive power is significantly improved. Normal sampling also leads to a more significant improvement in model prediction. It indicates that the importance of the sequential input structure in the absence of data is equivalent to that of the sampling layer, with the former giving the model the ability to adapt to the absence of data and the latter giving the model some imaginative power. Enabling HGP alone does not result in significant performance gains, but enabling both HGP and GPS together results in a significant performance improvement close to the performance of the component at full power. This result reveals the fundamental difference between GPS and normal sampling. GPS is sampling future observations of a Normal Stochastic Process, which corresponds to the complete normal distribution then and thus allows the model to adapt to incomplete data while forcing more attention and imagination to future information. On the other hand, GS only samples on a static normal distribution, which requires detailed input data. The sampling point is fixed at the point in time of the input information and cannot vary continuously along the time axis.

We also examine the effect of hyperparameter changes on the model. Latent time translation distance λ is responsible for defining the degree of time translation into the future in continuous virtual observations, and hyperparameters l and σ define the specific form of the normal kernel function in the HGP. We examine the effect of these parameter combinations on the performance of NNSP by setting different values of hyperparameters.

As seen in Table 4, when both hyperparameters of the kernel function are fixed, the model’s performance decreases as λ increases. As the latent time translation distance defines the foreseeable length of NNSP for future information. Intuitively, this changing relationship is easy to understand, i.e., predicting anomalous long-range information usually increases the difficulty of the model prediction. However, even if we enlarge λ to ten times the default value (λ = 10), the anomaly detection ability of the model does not decrease significantly (the F1 value decreases from 82.61% to 80.28%). The period of NNSP foreseeable for industrial time series at this point increased to 10 time steps, which can be considered as a limited decrease in anomaly detection accuracy in exchange for a more considerable increase in the foreseeable model period. It enables the model to adapt to industrial data anomaly detection tasks with high requirements for prediction timeliness.

## 5. Conclusions

To perform anomaly detection on incomplete nuclear power production monitoring data, we propose the NNSP model. Instead of relying on missing value imputation, NNSP directly transforms incomplete time series into continuous and complete latent representations using a Neural Normal Stochastic Process. The decoder samples future time points from these probabilistic latent representations to reconstruct samples that carry forward-looking information. By computing the reconstruction error, the model can efficiently detect anomalous states embedded in the input data.

Experimental results show that NNSP outperforms five mainstream baseline methods in anomaly detection tasks on incomplete nuclear monitoring data, achieving higher F1 scores and faster anomaly warning responses. Additionally, ablation studies confirm that the Neural Normal Stochastic Process is essential to the model’s performance. Parametric analysis further verifies the impact of latent time shift distance and kernel function settings on detection effectiveness.

Despite these promising results, several limitations remain. The relationship between the latent time shift distance, anomaly warning timeliness, and prediction accuracy requires further investigation. Moreover, the implementation of the Neural Normal Stochastic Process involves matrix inversion and repeated matrix multiplications, which results in high time complexity. Although such operations are well-suited to current GPU-based parallel computing environments, they may incur significant computational costs as the scale of sensor deployments and data volumes increases. Therefore, reducing the time complexity of the model will be a key focus of future work.

## Figures and Tables

**Table 1:** Dataset Specifications.

Strategy	PGS	PTS	PDS	TSS	EAL
Missing Rate	13.21%	11.17%	10.72%	9.66%	14.28%
Anomaly Rate	9.77%	14.02%	6.51%	12.41%	12.73%
Length	1695.25 K	712.33 K	37,286.04 K	1442.93 K	26,782.61 K
Frequency	1/30 s	1/60 s	1/s	1/30 s	1/1 s

**Table 2:** Anomaly detection (precision P/recall R/F1 as %).

Dataset	PGS			PTS			PDS			TSS			EAL		
Metric	P	R	F1	P	R	F1	P	R	F1	P	R	F1	P	R	F1
**ARMA**	58.42	54.13	56.19	64.29	57.80	60.87	60.64	50.44	55.07	55.75	53.39	54.55	62.94	52.10	57.12
**IsoForest**	65.49	67.89	66.67	67.50	74.31	70.74	66.36	62.83	64.55	66.06	61.02	63.44	57.26	64.11	60.51
**LSTM-AD**	73.12	62.39	67.33	75.45	76.15	75.80	73.87	72.57	73.21	71.43	67.80	69.47	68.32	63.59	65.90
**VAE**	77.32	68.81	72.82	75.26	66.97	70.87	69.37	68.14	68.75	71.68	68.64	70.13	70.74	65.16	67.86
**NeutraL AD**	84.16	77.98	80.95	79.21	73.39	76.19	78.07	78.76	78.41	82.35	71.19	76.36	80.53	74.27	77.27
**NNSP**	**84.91**	**82.57**	**83.72**	**83.33**	**81.67**	**82.49**	**84.26**	**80.53**	**82.35**	**85.19**	**77.97**	**81.42**	**83.70**	**79.94**	**81.78**

**Table 3:** Ablation results (F1 as %).

Strategy	PGS	PTS	PDS	TSS	EAL
SIS− HGP− GPS−	57.29	56.21	59.43	56.31	57.52
SIS+ HGP− GPS−	65.72	64.32	67.11	65.49	66.09
SIS+ HGP+ GPS−	69.23	68.97	67.87	66.15	67.49
SIS− HGP+ GPS−	65.85	62.71	63.34	65.92	62.24
SIS− HGP+ GPS+	77.97	76.47	69.83	70.20	73.76
SIS− HGP− GS+	72.82	70.87	68.75	70.13	68.18
SIS+ HGP− GS+	75.90	74.14	71.28	69.81	71.55
SIS+ HGP+ GPS+	83.72	82.49	82.35	81.42	81.78

**Table 4:** Hyperparameter variation (F1 as %).

Hyperparameters	F1			
	λ = 0.1	λ = 1	λ = 10	λ = 50
l = 1.0, σ = 1.0	84.29	82.61	80.28	76.73
l = 1.0, σ = 2.0	81.67	79.72	77.42	74.13
l = 0.5, σ = 1.0	79.96	78.38	75.02	73.29
l = 1.2, σ = 0.8	83.20	82.05	79.74	76.22

## Data Availability

The original contributions presented in this study are included in the article. Further inquiries can be directed to the corresponding author.
